# Impact of macular fluid features on outcomes of anti-vascular endothelial growth factor treatment for type 3 macular neovascularization

**DOI:** 10.1038/s41598-021-03053-w

**Published:** 2021-12-08

**Authors:** Wontae Yoon, Jihyun Yoon, Seung Kwan Na, Jihyun Lee, Jaemin Kim, Jong Woo Kim, Han Joo Cho

**Affiliations:** Kim’s Eye Hospital, Konyang University College of Medicine, 156, 4ga, Yeongdeungpo-dong, Yeongdeungpo-gu, Seoul, South Korea

**Keywords:** Retinal diseases, Outcomes research, Macular degeneration

## Abstract

We evaluated the impact of macular fluid features on visual and anatomical outcomes in type 3 macular neovascularization (MNV) patients treated with anti-vascular endothelial growth factor (VEGF). We retrospectively enrolled 89 eyes with type 3 MNV with at least 12 months of follow-up. All patients were treatment-naïve and received a monthly loading injection of anti-VEGF for three months, followed by further injections as required. The association of baseline macular morphology, including intraretinal fluid (IRF) and subretinal fluid (SRF), with visual and anatomical outcomes was analyzed. At baseline, IRF was present in all enrolled patients (100%), and SRF was present in 43.8% (39/89) of them. After 12 months of treatment, no significant difference was found in terms of best-corrected visual acuity (BCVA) and changes in central foveal thickness between the eyes with (39) and without (50) SRF at baseline. In addition, the proportion of improved or worsened (gain or loss of more than three lines in the BCVA) visual acuity at 12 months was not significantly different among the groups. Incidence of macular atrophy during the treatment showed no difference between the groups, regardless of the presence of SRF. In conclusion, the macular fluid morphology, specifically SRF, in type 3 MNV showed no significant correlation with visual and anatomical outcomes during anti-VEGF treatment.

## Introduction

Type 3 macular neovascularization (MNV) is a subtype of neovascular age-related macular degeneration (AMD), which was previously known as retinal angiomatous proliferation^1^. Despite controversies regarding the origin, recent evidence suggests that most of the type 3 MNV originate from the deep retinal plexus rather than from the choroid^2,3^. Recent clinicopathological investigations also support this hypothesis regarding the origin of type 3 MNV^3^. For over a decade, anti-vascular endothelial growth factor (anti-VEGF) therapy has been the standard treatment for type 3 MNV and other subtypes of neovascular AMD^4^. The long-term visual outcomes of anti-VEGF treatment in type 3 MNV are comparable to those in typical neovascular AMD^4^. However, development of macular atrophy is more frequent in type 3 MNV than in typical neovascular AMD^5^.

Optical coherence tomography (OCT) findings of the retinal fluid are among the major biomarkers for assessing disease activity in neovascular AMD^6^. On OCT, the fluid is identified as a hyporeflective lesion at various compartments including intraretinal, subretinal, and sub-retinal pigment epithelium (sub-RPE) spaces. Recently, many studies have reported that visual outcomes in neovascular AMD are affected by the type of fluid.

Post-hoc analyses of multiple major clinical trials including the Comparison of Age-Related Macular Degeneration Treatments (CATT) Trials^7^ and VEGF Trap-Eye: Investigation of Efficacy and Safety in Wet AMD (VIEW 2) trial^8^ have shown that intraretinal fluid (IRF) is associated with lower baseline visual acuity (VA) and worse visual outcomes when compared with subretinal fluid (SRF)^7,8^. Moreover, IRF has been reported to be associated with an increased risk of macular atrophy^7,9^. Consequently, there is a debate regarding whether residual SRF could be tolerated and monitored without aggressive anti-VEGF treatment^10^.

Although it is well known that fluid types at presentation are associated with visual outcomes after anti-VEGF treatment in neovascular AMD, the prognostic implications of fluid types in type 3 MNV have not been sufficiently reported. The purpose of this study was to identify the impact of macular fluid features on visual outcomes during anti-VEGF treatment in patients with type 3 MNV.

## Materials and methods

Data were collected retrospectively by screening the AMD database of Kim’s Eye Hospital. Patients diagnosed with and treated for type 3 MNV between January 2016 and October 2018 were screened for the analysis. The research followed the tenets of the Declaration of Helsinki and the Institutional Review Board at Kim’s Eye Hospital approved this study. The requirement for informed consent was waived by the Institutional Review Board at Kim’s Eye Hospital.

### Subjects

The inclusion criteria were cases of type 3 MNV confirmed with multimodal imaging including spectral domain OCT (SD-OCT), fluorescein angiography (FA), and indocyanine green angiography (ICGA) at the initial visit. Only patients who were treatment-naïve and had completed at least 12 months of follow-up were included. For cases of bilateral type 3 MNV, only the eye that was diagnosed first was included in the analysis. The exclusion criteria were end-stage conditions at presentation including disciform scars or fibrosis involving the fovea and cases with RPE tear at baseline or during the follow-up.

Type 3 MNV was diagnosed using a previously suggested method, based on the results of SD-OCT^2,11^. Additionally, FA/ICGA was used to confirm focal hyperfluorescence with late leakage from neovascularization. Type 3 MNV lesions were staged according to the recently suggested staging system using the SD-OCT findings^12^.

As a routine practice at Kim’s Eye Hospital, all eyes with neovascular AMD are treated with three monthly loading intravitreal injections (3 injections within 90 days of the first injection) of anti-VEGF agents (ranibizumab [0.5 mg/0.05 mL] or aflibercept [2 mg/0.05 mL]). After three initial injections, labeled usage, which is reimbursed through the South Korean National Health Insurance, consists of bimonthly injections of aflibercept and monthly injections of ranibizumab. Hence, after the initial loading injections, patients treated with the pro-re-nata (PRN) regimen were followed up at 4–8-week intervals during the study period. All patients underwent standardized examinations at every visit, including the best-corrected VA (BCVA), fundus examination, SD-OCT (consisting of 19 or 31 horizontal lines [6 mm × 6 mm area]), and additional FA/ICGA, OCT angiography, or autofluorescence (AF) at the physician’s discretion.

### Data analysis

The macular fluid features at baseline were evaluated by analyzing all OCT scans of the enrolled patients. Two independent retinal specialists (W.Y. and J.Y.) reviewed the OCT and FA/ICGA images to ensure uniform evaluation of all patients. IRF was defined as a hyporeflective space within the neurosensory retina on SD-OCT, excluding the spaces with a hyperreflective border that corresponded to outer retinal tubulation. The presence of persistent intraretinal cysts despite more than three consecutive anti-VEGF injections was considered as cystic degeneration (or pseudocyst) rather than IRF from the disease activity. In addition, it has been confirmed that cystic degeneration overlies diffuse RPE atrophy or scar tissue^13,14^. SRF was identified as a nonreflective space between the posterior boundary of the neurosensory retina and the RPE reflection on SD-OCT images.

Incident macular atrophy during anti-VEGF treatment was detected using color fundus photography, SD-OCT, infrared reflectance imaging, and AF, which was consistent with our previous investigations^5,11,15^. Macular atrophy was identified using the following criteria: (1) hypopigmented area greater than 250 µm within the macular vascular arcades, (2) hypofluorescence on AF images and increased visibility of the underlying choroidal vessels, and (3) confirmation of increased signal transmission in the choroid due to RPE atrophy on SD-OCT images.

The visual outcome was measured as the change in the BCVA (logarithm of the minimum angle of resolution [LogMAR] converted from Snellen BCVA) from baseline to 3, 6, 9, and 12 months. We also recorded the proportion of patients who exhibited a gain or loss of more than three lines in the BCVA compared to the baseline.

In case of disagreement between the graders regarding the staging, evaluation of the OCT images, or identification of macular atrophy, the case was reviewed by the senior principal investigator (H.J.C.) and the final determination was made after an open discussion.

### Statistical analysis

Results were expressed as means and standard deviations for continuous variables. Frequencies were compared between the groups using the chi-squared test or Fisher’s exact test. Comparative statistical analyses were performed using unpaired *t*-tests. SPSS software version 18.0 (SPSS Inc., Chicago, IL, USA) was used for all statistical analyses and *P* < 0.05 was considered statistically significant.

## Results

Altogether, 125 eyes having type 3 MNV and treated with anti-VEGF therapy were initially screened during the study period. Among these, 36 eyes were excluded for the following reasons: 10 eyes were excluded due to development of RPE tear at baseline or during the study period, six eyes were excluded due to end-stage conditions such as foveal geographic atrophy or scar at presentation, and 20 eyes were excluded, as these patients did not complete the 12-month follow-up. Thus, 89 eyes were included in the analysis.

All patients were South Korean and the mean age of the entire study group was 78.1 ± 7.1 years. The mean number of anti-VEGF injections was 5.1 ± 2.4 during the 12-month study period (range 3–9). At baseline, IRF was present in all enrolled subjects (100%), and SRF was present in 43.8% (39/89) of them. The clinical details of the patients included in this study are presented in Table 1. When comparing the various characteristics between the groups with and without SRF at baseline, no significant difference was found regarding age, sex, baseline BCVA, baseline central foveal thickness, subfoveal choroidal thickness, stage of type 3 neovascularization, presence of reticular pseudodrusen, and anti-VEGF agents (Table 1).

**Table 1:** Baseline characteristic of patients with type 3 macular neovascularization.

	Eyes with type 3 macular neovascularization (n = 89)	SRF (−) at baseline (n = 50)	SRF (+) at baseline (n = 39)	*P*
**Age in years, mean ± SD**	78.1 ± 7.1	78.4 ± 8.1	77.9 ± 7.8	0.668^a^
**Sex, n (%)**				0.356^b^
Male	34 (38.2%)	17 (34.0%)	17 (43.6%)	
Female	55 (61.8%)	33 (66.0%)	22 (56.4%)	
**Baseline BCVA in LogMAR, mean ± SD (Snellen equivalent)**	0.55 ± 0.41 (20/70)	0.54 ± 0.47 (20/69)	0.58 ± 0.40 (20/76)	0.521^a^
**Baseline BCVA in LogMAR (Snellen equivalent), n (%)**				0.853^b^
< 0.40 (20/50)	33 (37.1%)	18 (36.0%)	15 (38.5%)	
0.40 (20/50) to 1.0 (20/200)	45 (49.4%)	26 (52.0%)	19 (48.7%)	
> 1.0 (20/200)	11 (13.5%)	6 (12.0%)	5 (12.8%)	
**Baseline central foveal thickness (µm), mean ± SD**	339 ± 127	321 ± 122	352 ± 145	0.365^a^
**Subfoveal choroidal thickness (µm), mean ± SD**	148 ± 57	150 ± 55	145 ± 59	0.557^a^
**Stage of type 3 neovascularization, n (%)**^**c**^				0.098^c^
Stage 1	5 (5.6%)	5 (10.0%)	0 (0%)	
Stage 2	13 (14.6%)	9 (18.0%)	4 (10.3%)	
Stage 3	71 (79.8%)	36 (72.0%)	35 (89.7%)	
**Presence of reticular pseudodrusen at baseline, n (%)**	63 (70.8%)	34 (68%)	29 (74.4%)	0.513^b^
**Anti-VEGF agent, n (%)**				0.639^b^
Ranibizumab	39 (43.8%)	23 (46.0%)	16 (41.0%)	
Aflibercept	50 (56.2%)	27 (54.0%)	23 (59.0%)	

*BCVA* best-corrected visual acuity, *IRF* intraretinal fluid, *LogMAR* logarithm of the minimum angle of resolution, *PRN* pro-re-nata, *RPE* retinal pigment epithelium, *SD* standard deviation, *SRF* subretinal fluid, *TNE* treat-and-extend, *VEGF* vascular endothelial growth factor.

^a^*P*-value by t-test.

^b^*P*-value by chi-square test.

^c^*P*-value by Fisher’s exact test.

**Figure 1:**
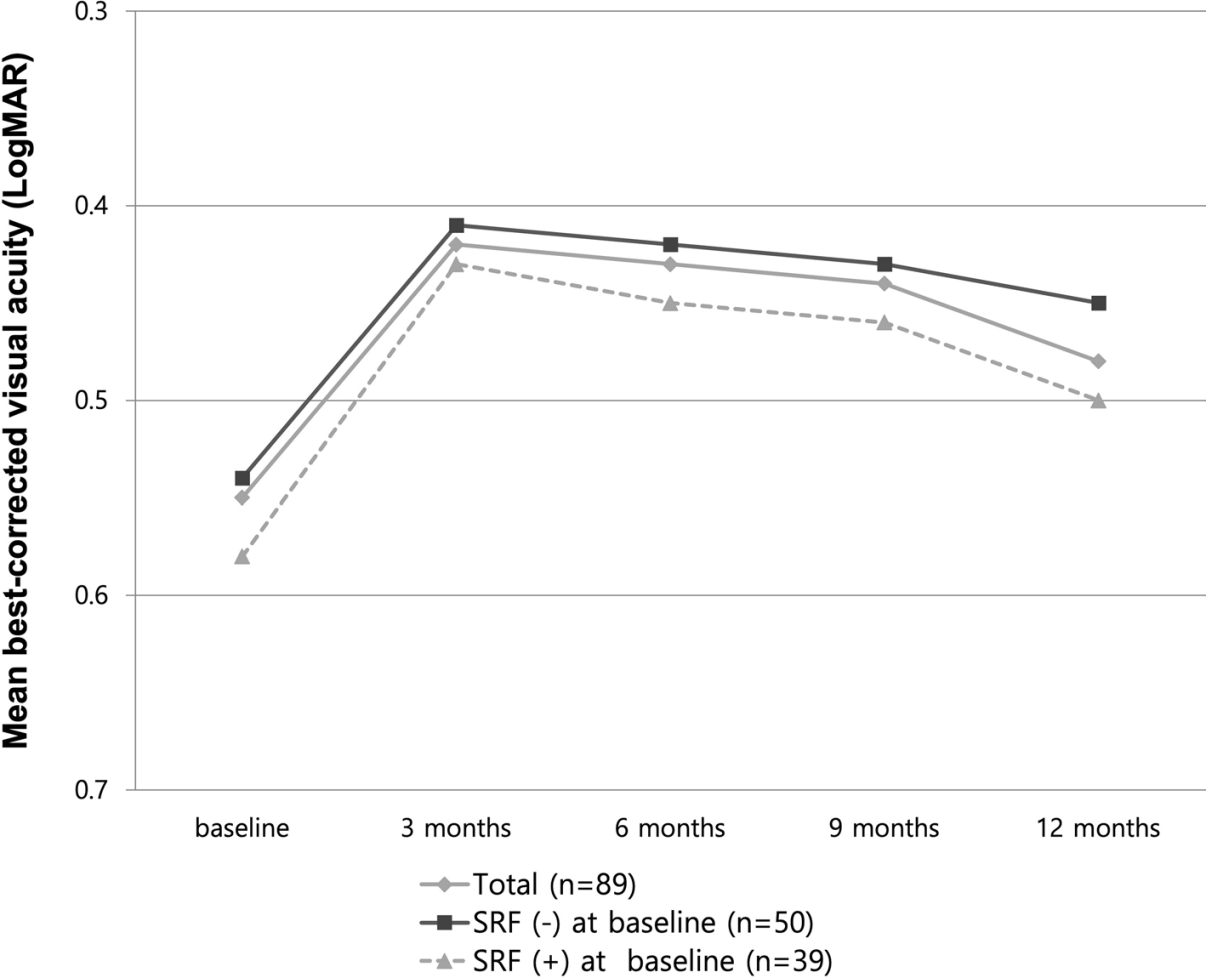
Changes in the mean best-corrected visual acuity (BCVA) are expressed as the logarithm of the minimum angle of resolution (LogMAR) during the 12-month follow-up of patients treated with anti-vascular endothelial growth factor for type 3 macular neovascularization. The graph shows the BCVA changes in all eyes, in eyes with, and without subretinal fluid (SRF) at baseline. No statistically significant difference was observed at 3, 6, 9, and 12 months among the groups (*P* = 0.636, *P* = 0.512, *P* = 0.711, and *P* = 0.627; respectively).

The mean BCVA of all the eyes improved from 0.55 ± 0.41 LogMAR (Snellen equivalent: 20/70) at baseline to 0.48 ± 0.37 LogMAR (20/60, *P* = 0.012) at 12 months (Fig. 1). The BCVA changes in eyes with (39) and without (50) SRF at baseline were similar to those in the total subjects. The BCVAs improved from 0.58 ± 0.40 LogMAR (20/76) at baseline to 0.50 ± 0.39 LogMAR (20/63) at 12 months (*P* = 0.019) in eyes with SRF, and from 0.54 ± 0.47 LogMAR (20/69) at baseline to 0.45 ± 0.36 LogMAR (20/56) at 12 months (*P* = 0.006) in eyes without SRF. There was no significant difference between the groups at 3, 6, 9, and 12 months (*P* = 0.636, *P* = 0.512, *P* = 0.711, and *P* = 0.627, respectively; Fig. 1).

**Figure 2:**
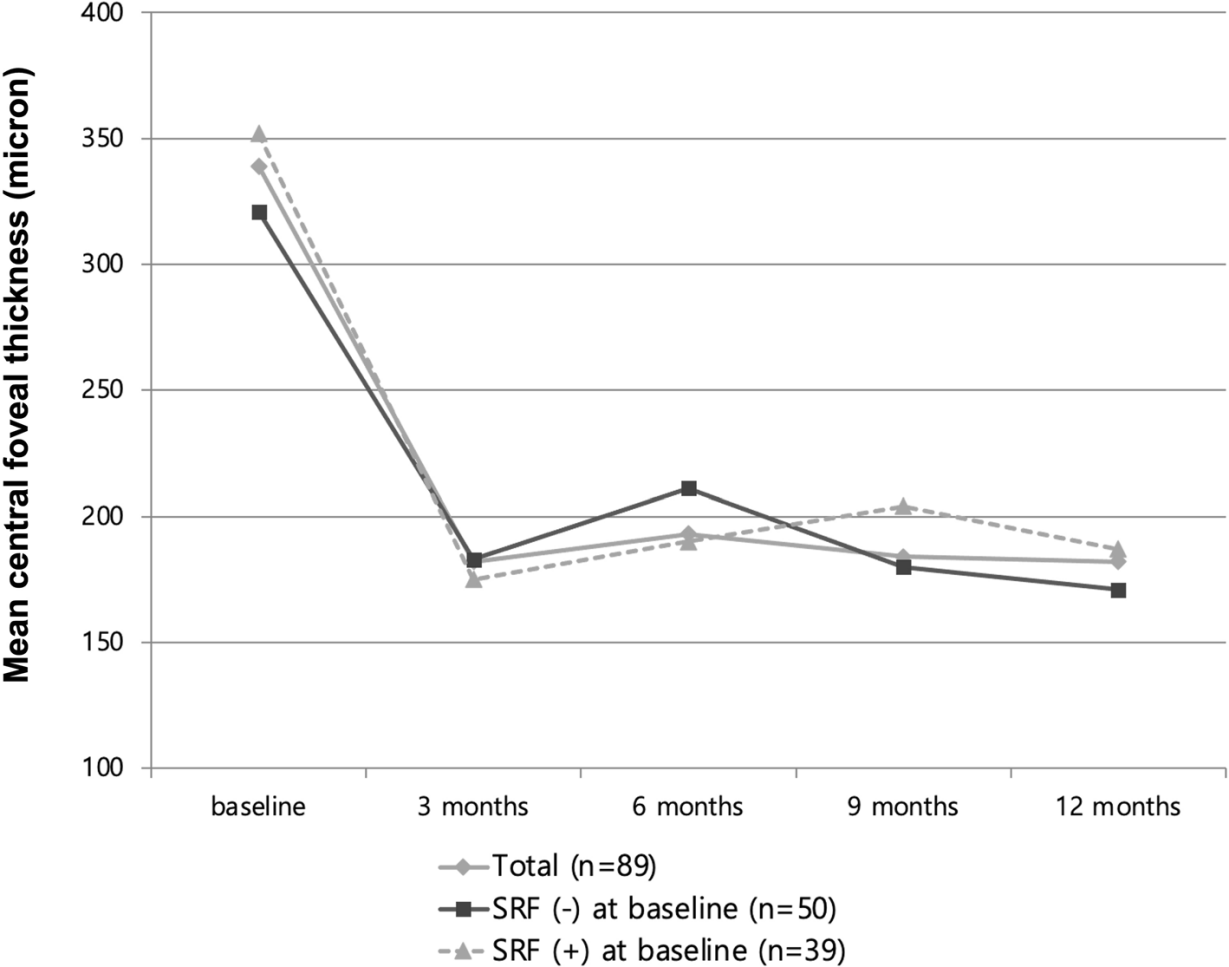
Changes in central foveal thickness during the12-month follow-up of patients treated with anti-vascular endothelial growth factor for type 3 macular neovascularization. No significance difference was observed among the subgroups including eyes with and without subretinal fluid (SRF) at 3, 6, 9, and 12 months (*P* = 0.455, *P* = 0.627, *P* = 0.337, and *P* = 0.561, respectively).

The mean central foveal thickness showed comparable changes between the groups (Fig. 2). In cases of eyes without SRF, it significantly improved from 321 ± 122 µm at baseline to 171 ± 153 µm at 12 months (*P* = 0.003), while eyes with SRF also showed a significant improvement from 352 ± 145 µm at baseline to 187 ± 143 µm at 12 months (*P* = 0.018). However, no significant intergroup difference was observed at 3, 6, 9, and 12 months (*P* = 0.455, *P* = 0.627, *P* = 0.337, and *P* = 0.561, respectively; Fig. 2).

After 12 months of anti-VEGF treatment, the proportion of improved (a gain of ≥ three lines in the BCVA) and worsened (a loss of ≥  three lines in the BCVA) visual acuity was not significantly different with respect to the fluid features (Table 2). The proportion of eyes with improved visual acuity was 26.0% among eyes without SRF and 30.7% among eyes with SRF, showing no statistically significant difference between the groups (*P* = 0.619, Table 2). Moreover, the proportion of eyes with worsened visual acuity was 18.0% among eyes without SRF and 23.1% among eyes with SRF, also showing no statistically significant difference among the groups (*P* = 0.554, Table 2).

**Table 2:** One-year results of treatment for type 3 macular neovascularization according to the baseline fluid features.

	Total (89 eyes)	Baseline fluid feature		*P*
		SRF (−) (50 eyes)	SRF (+) (39 eyes)	
**BCVA in LogMAR at 12 months, mean ± SD (Snellen equivalent)**	0.48 ± 0.37 (20/60)	0.45 ± 0.36 (20/56)	0.50 ± 0.39 (20/63)	0.213^a^
**Mean BCVA change in LogMAR from baseline**	− 0.11	− 0.12	− 0.08	0.112^a^
**BCVA changes, n (%)**				
Improved ≥ LogMAR 0.3	25 (28.1%)	13 (26.0%)	12 (30.7%)	0.619^b^
Worsened ≥ LogMAR 0.3	18 (20.2%)	9 (18.0%)	9 (23.1%)	0.554^b^
**Changes in mean central foveal thickness from baseline, µm**	− 157	− 150	− 165	0.611^a^
**Changes in mean subfoveal choroidal thickness from baseline, µm**	− 14	− 13	− 15	0.714^a^
**Macular atrophy, n (%)**	30 (33.7%)	14 (28.0%)	16 (41.0%)	0.197^b^
**Number of anti-VEGF injections, mean ± SD**	5.1 ± 2.4	4.8 ± 2.3	5.4 ± 2.8	0.138^a^

*BCVA* best-corrected visual acuity, *IRF* intraretinal fluid, *LogMAR* logarithm of the minimum angle of resolution, *RPE* retinal pigment epithelium, *SD* standard deviation, *SRF* subretinal fluid, *VEGF* vascular endothelial growth factor.

^a^*P*-value by t-test.

^b^*P*-value by chi-square test.

Macular atrophy developed in 33.7% (30/89) of the enrolled eyes during the 12-month anti-VEGF treatment. The incidence of macular atrophy during the study period tended to be higher among eyes with IRF and SRF (41.0%) than in IRF only group (28.0%). However, no statistically significant differences were observed among the groups (*P* = 0.197, Table 2). During the 12 months anti-VEGF treatment, degenerative cysts were identified in 6.7% (6/89) of the total cases. The incidence of degenerative cysts showed no differences between the eyes with IRF and SRF (6.0% [3/50]) and the IRF only group (7.7% [3/39], *P* = 0.752).

The mean number of injections during the 12-month treatment was 4.8 ± 2.3 for eyes without SRF, and 5.4 ± 2.8 for eyes with SRF, with no significant difference observed among the groups (*P* = 0.138, Table 2).

## Discussion

It is well known that IRF in neovascular AMD is associated with lower baseline VA, delayed response to anti-VEGF treatment, and poor visual outcome^16^. On the other hand, SRF is associated with better visual outcome, lower frequency of anti-VEGF injections than that in patients with IRF^10^, and lower frequency of development of macular atrophy^17^. However, these correlations between visual outcome and features of macular fluid were not observed in our cohort of patients with only type 3 MNV.

Type 3 lesions have been proposed to originate from the deep capillary plexus rather from than the choroid and descend to the abutting RPE as they progress^2,18^. The development of neovascularization from the neurosensory retina results in IRF in the early stage. Consequently, IRF is observed in almost every case of type 3 MNV not only at presentation but also at recurrence^4,19^. In the present study, the incidence of SRF at baseline (43.8%) was lower than that reported in previous studies (84.1% in the VIEW 2 study and 83.2% in the Tolerating Subretinal Fluid in Neovascular Age-Related Macular Degeneration Treated with Ranibizumab Using a Treat-and-Extend Regimen [the FLUID study]), which included mainly type 1 or type 2 MNV^10,14^. Our results suggest that the baseline distribution of macular fluid in type 3 MNV is different from that in other types of neovascular AMD.

Disruption of the outer blood-retinal barrier due to active MNV results in exudation into the subretinal space (SRF) and disruption of the external limiting membrane can cause accumulation of fluid in the neurosensory retina (IRF)^20^. Alteration of bipolar axons by intraretinal cystic fluid results in neurosensory damage^21^. Importantly, the neurosensory damage caused by IRF cannot be reversed by additional anti-VEGF treatment^7^. Quantitative studies have demonstrated that approximately 20% of VA outcomes are already determined by irreversible cyst-mediated neurosensory damage at baseline irrespective of the intensity of anti-VEGF therapy^22^. Therefore, it has been proposed that IRF should not be tolerated and should be treated more aggressively than SRF in neovascular AMD^14,23^.

Although IRF, a well-known predictive factor for poor prognosis, is nearly always present in type 3 MNV, the visual outcomes after anti-VEGF treatment in type 3 MNV are not worse and are even comparable or better than those in other types of neovascular AMD^4,24^. In the present study, the visual outcomes were not associated with the presence of IRF at baseline. Furthermore, the presence of SRF at baseline was not associated with better visual outcomes after anti-VEGF treatment in our study. Our results suggest that the implications of IRF or SRF with respect to visual prognosis in type 3 MNV might be different from those in other types of neovascular AMD.

It is uncertain why the visual outcomes in type 3 MNV are not significantly affected by the fluid features. However, several reasons could be attributed to the characteristics of type 3 MNV. Type 3 MNV is generally smaller^24^, resulting in rather minute exudation compared to other types of neovascular AMD^18,25^. In addition, the exudation and PED in type 3 lesions usually respond rapidly to treatment, since type 3 lesions are more sensitive to anti-VEGF therapy compared to other types of neovascular AMD^11,24^. Furthermore, IRF in type 3 MNV is related to increased tissue VEGF levels and not necessarily to neovascularization alone^26^. Hence, the visual outcomes in type 3 MNV could be less affected by IRF than those in other types of neovascular AMD. Further investigations are warranted to determine why the fluid features in type 3 MNV showed no definite correlation with the visual outcomes.

The presence of SRF is associated with a decreased incidence of macular atrophy in neovascular AMD^7,9,17^. However, in the present study involving only type 3 MNV, the incidence of macular atrophy was not affected by the presence of SRF. This finding could be associated with the higher incidence and faster growth of macular atrophy in patients with type 3 MNV compared to other types of MNV^5,11,27^. Type 3 MNV is characterized by a thin choroid and impaired choriocapillaris perfusion, which causes vulnerability to RPE atrophy^5,28^. Thus, macular atrophy in type 3 MNV could be affected by the characteristic choroidal perfusion state rather than by macular fluid condition resulting from the neovascularization. Additional investigations need to be conducted in the future to determine the exact association between macular fluid and macular atrophy in type 3 MNV.

Our study has several limitations, in addition to its retrospective nature. First, we could not evaluate the association between the presence of IRF at baseline and treatment outcomes because all cases of type 3 MNV showed IRF at baseline. Considering that recent studies quantifying IRF in three-dimensional SD-OCT have reported that the amount of IRF is correlated with visual acuity^29^, the correlation between the IRF in type 3 MNV and visual outcomes should be investigated quantitatively in the future. Second, patients receiving the two anti-VEGF agents were not strictly differentiated. However, our results would not be significantly affected because it has been reported that ranibizumab and aflibercept are equally effective in the management of type 3 MNV^11^. Third, our results could be different under more frequent, intensive injection treatments, similar to monthly fixed injections used in clinical trials.

In conclusion, the macular fluid features in type 3 MNV showed no significant correlation with visual outcomes. The presence of IRF or SRF at baseline was not associated with the incidence of macular atrophy in type 3 MNV. These results relatively vary from previous reports on the association between macular fluid features and prognosis in other subtypes of neovascular AMD. Further investigations regarding the reasons behind these findings in type 3 MNV and their clinical significance are warranted.
